# Does Social Media Use Associate with Vasomotor, Sexual, and Musculoskeletal Symptoms in Breast Cancer Survivors Receiving Endocrine Therapy?

**DOI:** 10.3390/jcm15124726

**Published:** 2026-06-18

**Authors:** Halil Göksel Güzel, Ece Ulukal Karancı, Derya Kıvrak Salim, Murat Koçer, Banu Öztürk

**Affiliations:** 1 Department of Medical Oncology, Antalya City Hospital, Gocerler, 5379 St., Kepez, 07080 Antalya, Turkey; 2 Department of Medical Oncology, Antalya Training and Research Hospital, Varlik, Kazım Karabekir St., Muratpasa, 07080 Antalya, Turkey

**Keywords:** breast cancer survivorship, endocrine therapy, patient-reported outcomes, social media use, digital behaviors

## Abstract

**Purpose:** Vasomotor, sexual, and musculoskeletal symptoms are common adverse effects of adjuvant endocrine therapy in breast cancer survivors. Social media use has not been investigated with altered symptom perception in patients receiving adjuvant endocrine therapy. This study aimed to investigate whether social media use or addiction independently predicts endocrine therapy-related symptom burden in breast cancer survivors. **Methods:** A cross-sectional survey study was conducted among 153 breast cancer survivors receiving adjuvant endocrine therapy. The Social Media Use Scale (SMUS) and Bergen Social Media Addiction Scale (BSMAS) were assessed using validated Turkish versions of each scale. Endocrine therapy-related toxicities (specifically hot flashes, vaginal dryness, loss of libido, and musculoskeletal pain severity) were evaluated using specific self-reported 5-point Likert scale items. **Results:** All of the patients were female and menopausal, either neutral or induced with ovarian function suppression. In the univariate analysis, the BSMAS score showed a weak positive correlation with vasomotor/sexual symptoms (r = 0.194; *p* = 0.017), but this association disappeared after adjustment for clinical variables. Younger age was associated with greater vasomotor/sexual symptoms in univariate testing. Neither the SMUS nor BSMAS independently predicted musculoskeletal symptom severity in univariate and multivariate models, while higher educational attainment remained the only independent predictor of musculoskeletal pain severity (OR = 1.96; 95% CI: 1.06–3.57; *p* = 0.031). **Conclusions:** This study is unique in investigating unstructured social media use and endocrine therapy-related physical symptoms. In this cohort, unstructured social media use was not associated with the endocrine therapy-related physical symptom burden. While these cross-sectional findings do not support social media behavior as a significant predictor, clinical assessments should continue to prioritize established determinants such as age and educational background.

## 1. Introduction

Early-stage hormone receptor-positive breast cancer accounts for up to 80% of all breast cancer diagnoses, and adjuvant endocrine therapy (aromatase inhibitors in postmenopausal women; tamoxifen ± ovarian suppression in premenopausal patients) remains a cornerstone for reducing recurrence and improving long-term survival [1,2,3]. However, long-term endocrine therapy is frequently accompanied by a spectrum of adverse events with characteristic symptoms. These adverse symptoms can be classified as vasomotor symptoms (e.g., hot flashes, night sweats), sexual dysfunction (e.g., vaginal dryness, decreased libido), and musculoskeletal pain [3,4].

Vasomotor, sexual, and musculoskeletal symptoms experienced during adjuvant endocrine therapy are inherently subjective and often overlap with manifestations of anxiety and depression, both in pathophysiology and in patient-reported perception [5]. Concurrently, intense or dysregulated social media use has been increasingly linked to adverse psychological outcomes, particularly anxiety, depressive symptoms, sleep impairment, and diminished stress tolerance [6,7]. However, while the impact of digital behaviors on these psychosocial outcomes is well-documented, the degree to which they translate into altered somatic symptom perception remains largely unexplored and partly speculative. In the context of endocrine therapy-related toxicities, structured mindfulness-based interventions and yoga have demonstrated that targeted behavioral habits can modulate symptom perception [8]. Moreover, the broader literature indicates that patterns of social media engagement can influence psychological well-being, stress, sleep, and pain sensitivity [9]. Within the breast cancer population, app-based or digitally delivered supportive interventions have demonstrated meaningful improvements in psychosocial and functional outcomes, reinforcing the notion that digital platforms may modulate patient experiences in both beneficial and detrimental ways, depending on the utilization [10]. While these findings indicate that structured digital platforms can positively guide patient experiences, it remains unclear whether unstructured, daily social media engagement shares a similar regulatory or detrimental relationship with somatic symptom reporting.

Despite these developments, a marked gap exists in the literature regarding whether social media behavior is associated with endocrine therapy-related physical symptoms in breast cancer survivors. Most current studies focus on psychosocial outcomes, coping strategies, or quality of life in broad terms, rather than investigating the interactions between behavioral (social media) variables and the somatic adverse effects of endocrine therapy [11,12,13]. While symptom cluster analyses have identified key predictors of therapy intolerance and non-adherence, social media use and addiction have not been investigated as potential modifiable factors. Therefore, this study was designed to address the unexplored association between digital behavior and endocrine treatment toxicity in breast cancer survivors.

This study aimed to evaluate the association between social media use metrics (usage level and addiction tendency) and the burden of endocrine therapy-related vasomotor/sexual and musculoskeletal symptoms, thereby providing a foundational baseline for understanding digital behaviors within this patient population.

## 2. Material and Methods

### 2.1. Study Population and Survey Instruments

This was a single-center cross-sectional survey-based study conducted at the Department of Medical Oncology of Antalya Training and Research Hospital, between 7 July 2025 and 11 October 2025. Written and verbal informed consent was obtained from all participants before enrollment. All patients received treatment in accordance with contemporary clinical guidelines during the study period.

All study procedures were performed in accordance with the ethical principles of the Declaration of Helsinki. The study protocol received approval from the Antalya Training and Research Hospital Scientific Research Ethics Committee on 3 July 2025 (Approval No: 2025-215, Decision No: 11/11).

The inclusion criteria were as follows:

1. Having a diagnosis of non-metastatic, hormone receptor-positive invasive breast cancer
2. Completion of curative-intent primary treatment
3. Currently receiving adjuvant endocrine therapy (aromatase inhibitor for postmenopausal patients, or tamoxifen plus active ovarian function suppression [OFS] for premenopausal patients)
4. Age ≥ 18 years,
5. Ability to provide written and verbal informed consent

The exclusion criteria were as follows:

1. Diagnosis of metastatic breast cancer
2. Presence of a second primary malignancy
3. Premenopausal status without active ovarian function suppression (OFS)
4. Complete absence of social media use, defined as not using any social media platform for personal or professional purposes
5. Incomplete follow-up data or inability to retrieve clinical information from hospital records

Participants completed two Turkish versions of validated questionnaires: the Bergen Social Media Addiction Scale (BSMAS) [14,15] and the Social Media Use Scale (SMUS) [16,17], both of which were translated from their original forms and psychometrically validated to assess social media use patterns and addiction. The BSMAS consists of six items rated on a 5-point Likert scale, while the SMUS includes ten items, and performed on a 6-point Likert scale, one of which is reverse-coded. Because general social media use does not necessarily indicate addictive use, and addiction represents a distinct behavioral construct, the SMUS (use level) and BSMAS (addiction tendency) scores were analyzed separately. The mean BSMAS and SMUS scores were calculated for each patient.

Complete absence of social media use, defined as not using any social media platform for personal or professional purposes. Exclusion of these patients was methodologically necessary because individuals with zero exposure cannot evaluate items on the BSMAS and SMUS.

For the BSMAS and SMUS questionnaires, missing data were managed using an available-case approach for each questionnaire. Accordingly, participants with two or more missing items were excluded from the score calculation, whereas a single missing item was tolerated, and the mean score was calculated using the available response.

To capture the direct somatic severity of targeted endocrine toxicities while minimizing participant response burden and survey fatigue in an outpatient oncology setting, brief investigator-developed Likert items were utilized instead of extensive quality-of-life questionnaires. To assess vasomotor and sexual symptoms, patients rated the severity of hot flashes, vaginal dryness, and loss of libido using a 5-point Likert scale (1 = never, 5 = very much). The three items were summed to generate the Vasomotor/Sexual Symptom Score. Patients who left two or more of these three items unanswered were excluded from the Vasomotor/Sexual Symptom Score analysis but were included in other analyses when applicable. The internal consistency of the 3-item Vasomotor/Sexual Symptom Score was acceptable, with a Cronbach’s alpha of 0.764 (standardized α = 0.765), indicating good reliability. Musculoskeletal pain was assessed using a single 5-point Likert item (1 = never, 5 = very much). Patients who did not answer this item were excluded from the Musculoskeletal Pain Score analysis, although they were included in other analyses whenever possible. Musculoskeletal pain responses were treated as ordinal variables in subsequent analyses.

A total of 176 patients were initially screened for their eligibility. Among them, 17 patients reported that they did not use social media. An additional 6 patients did not provide a sufficient number of valid responses to the BSMAS and SMUS questionnaires to allow the calculation of the respective scores. Consequently, the final analyses were conducted on 153 patients (*n* = 153).

### 2.2. Data Collection

Clinical data of the study population were obtained from patient follow-up charts and the hospital’s electronic medical record system. The following variables were recorded for each participant: age, sex, ECOG PS (Eastern Cooperative Oncology Study Group Performance Status), chemotherapy history, estrogen and progesterone receptor status (ER/PR), HER2 status, adjuvant endocrine therapy type (as tamoxifen or aromatase inhibitor), Tumor, Node, and Metastasis (TNM) stage.

TNM Staging was performed using the American Joint Committee on Cancer (AJCC) TNM Staging 7th Edition for patients treated before 2017 and the AJCC TNM Staging 8th Edition for those treated in 2017 or later.

### 2.3. Statistical Analyses

Statistical analyses were performed using IBM SPSS Statistics for Windows, Version 24.0. Continuous variables were expressed as mean ± standard deviation (SD) or median (min-max), and categorical variables were expressed as frequencies and percentages.

The normality of continuous variables was assessed using visual inspection of histograms and Q–Q plots, as well as the Shapiro–Wilk test. For correlation analyses, Pearson’s correlation was used for normally distributed variables, whereas Spearman’s rank correlation was applied when the assumption of normality was not met. The correlation coefficient is denoted as r. For comparisons between two independent groups, the independent samples *t*-test was used for normally distributed data, whereas the Mann–Whitney U test was applied for non-normally distributed data.

The vasomotor/sexual symptom score, a continuous variable, was further analyzed using multivariate linear regression. Variables with a univariate *p* < 0.3 and those considered clinically relevant were included in the multivariate model. Unstandardized regression coefficients (B) and 95% confidence intervals (CIs) were reported. The musculoskeletal pain score was based on a 5-point Likert scale and treated as an ordinal dependent variable. Therefore, multivariate modeling was performed using ordinal logistic regression (proportional odds model). The results were expressed as odds ratios (ORs) with 95% CI. A two-sided *p*-value < 0.05 was considered to be statistically significant.

## 3. Results

### 3.1. Study Population Characteristics

A total of 153 patients were available for the final analyses (*n* = 153). The median duration of endocrine therapy use was 2.23 years (0.05–9.48). The median number of social media platforms used was 2 (1–4). Most patients were postmenopausal (*n* = 97, 63.4%), and all received an aromatase inhibitor (AI) as endocrine therapy. The remaining 36.6% (*n* = 56) were premenopausal and were treated with ovarian function suppression (OFS) using LHRH analogs combined with tamoxifen (TMX). The detailed demographic and clinicopathological characteristics are summarized in Table 1.

### 3.2. Univariate and Multivariate Analyses for Vasomotor/Sexual Symptom Score

The vasomotor/sexual symptom score was calculated for 151 patients, as two patients did not answer the appropriate number of survey questions. The median value for vasomotor/sexual symptom score was 3.00 (1.00–5.00).

In the univariate analyses, there was a weak but statistically significant positive correlation between the vasomotor/sexual symptom score and the BSMAS score (**r = 0.194, *p* = 0.017**), whereas the correlation with the SMUS score was not significant (r = 0.083, *p* = 0.310). A moderate and statistically significant negative correlation was observed between age and the vasomotor/sexual symptom score (**r = −0.360, *p* < 0.001**), indicating that younger patients reported higher symptom levels.

In the multivariate linear regression analysis, age was not a significant predictor of the vasomotor/sexual symptom score. Neither the BSMAS nor the SMUS scores had a significant effect after adjusting for other covariates. Marital, educational, and working status; HER2 status; endocrine therapy type; chemotherapy history; and nodal status were not statistically significant in the multivariate model.

In summary, while the BSMAS score demonstrated a statistically significant correlation with vasomotor/sexual symptoms in univariate analysis, this correlation was weak in magnitude (r = 0.194) and did not remain a robust predictor after adjusting for clinical covariates. No independent predictors of the vasomotor/sexual symptom score were identified in the final multivariate regression model (Table 2).

### 3.3. Univariate and Multivariate Analyses for Musculoskeletal Pain Score

The musculoskeletal pain score was calculated for 152 patients, as one patient did not provide a valid response to the relevant survey item. The median musculoskeletal pain score was 3.00 (1.00–5.00).

No significant correlations were observed between the musculoskeletal pain score and either the BSMAS (r = 0.053, *p* = 0.515) or SMUS (r = 0.064, *p* = 0.434) scores. Age demonstrated a weak negative correlation with musculoskeletal pain (**r = −0.185, *p* = 0.022**); however, this association did not persist in the multivariate analysis (OR = 0.98; 95% CI: 0.95–1.01; *p* = 0.149). Educational status was the other variable that showed a significant association in the univariate analysis (***p* = 0.026**). After adjusting for covariates, higher educational attainment remained independently associated with increased odds of reporting greater musculoskeletal pain severity (**OR = 1.96; 95% CI: 1.06–3.57; *p* = 0.031**).

Synthesizing the multivariate ordinal logistic regression model for musculoskeletal pain, higher educational attainment emerged as the sole independent predictor of greater symptom severity. Neither age nor the social media metrics (BSMAS and SMUS) retained any significant independent predictive value after multivariable adjustment (Table 3).

## 4. Discussion

In this study, we comprehensively evaluated the association between social media use and two clinically relevant symptom clusters, vasomotor/sexual symptoms and musculoskeletal pain, in early-stage hormone receptor-positive breast cancer survivors undergoing adjuvant endocrine therapy. Our findings indicate that neither general social media use patterns (SMUS) nor social media addiction tendencies (BSMAS) independently predicted symptom severity in the multivariate analyses. Although a weak positive correlation between BSMAS scores and vasomotor/sexual symptoms emerged in univariate testing, this association was attenuated after adjustment for key demographic and clinical covariates, underscoring the multifactorial nature of symptom perception during endocrine therapy. Similarly, musculoskeletal pain, another frequently reported therapy-related adverse effect, was not associated with either social media metrics. Within the constraints of this specific cohort, these findings indicate that varying intensities of unstructured social media use were not significantly associated with endocrine therapy-related physical symptom burden. However, due to the study’s cross-sectional design and the exclusion of non-users, this lack of statistical correlation should be interpreted as an absence of observed evidence in this sample rather than definitive proof that digital behavior exerts no biological or psychological effect.

Our study revealed no association between social media use habits and symptoms associated with adjuvant endocrine therapy in breast cancer survivors. Theoretically, several psychophysiological mechanisms support a potential link between social media engagement and the perception of endocrine therapy-related somatic symptoms [6,7]. Because our study did not directly measure baseline psychiatric diagnoses, coping mechanisms, or objective stress parameters, these broader psychosocial frameworks must be explicitly delineated as hypothesis-generating contexts rather than direct explanatory mechanisms for our current empirical data. Although we did not adjust our study variables for psychiatric disorders, it is well known that breast cancer survivors face many psychosocial, emotional, sexual, and financial challenges that significantly impact their quality of life [18]. In one study, the depression rate was reported to be as high as 32.2% among patients with breast cancer [19]. These psychological challenges were associated with significant increases in social media use, sexual dysfunction, and musculoskeletal pain [20,21,22,23]. Social media has become a tool for self-management, access to tailored support, navigating identity changes, and regaining a sense of agency during and after breast cancer treatment [24]. Beyond the social role, patients with breast cancer have higher levels of online information-seeking and engagement than individuals with other cancer types [25]. Social media serves as a flexible and inclusive conduit for medical information exchange, offering patients access to educational materials in diverse formats such as videos, animations, and images. These contents could help with self-identification of the endocrine therapy adverse events and seeking for professional help [26]. Social media communities promote ongoing emotional support through shared patient narratives, and open communication about treatment-related challenges [27]. Anonymity on social media was shown to allow cancer patients to discuss sensitive subjects such as sexual symptoms without promoting self-disclosure [28,29]. These benefits may potentially help patients to handle and/or recognize endocrine therapy adverse effects. However, conflicting results have been reported regarding their clinical implications. The positive effects of social media were confirmed in a cross-sectional study by Farpour et al., who demonstrated that social media use was associated with better general health quality. They also reported that age was not associated with general health quality, whereas a positive history of chemotherapy was detrimental [30]. Although these results are inconsistent with our results, our data focused on the adverse effects of endocrine therapy rather than general health quality. In contrast, Moorhead et al. demonstrated that social media platforms often lack adequate quality control, leading to potential breaches of confidentiality, misinformation, and information overload that may lead to increased anxiety and abnormal perceptions of adverse events [31]. Moreover, unverified claims and “alternative” cancer remedies such as supplements, diets, or devices promoted on social media may alter endocrine therapy efficacy and adverse events [32].

The persistent association between higher educational attainment and increased musculoskeletal pain severity in our multivariate model warrants further attention. This finding is consistent with prior research suggesting that individuals with higher education often exhibit increased health awareness, heightened symptom appraisal, and a lower threshold for reporting functional limitations [33]. Another explanation may involve occupational patterns, since highly educated individuals may engage in more sedentary desk-based activities that predispose them to musculoskeletal pain, independent of endocrine therapy effects [34]. However, because our database lacked explicit parameters tracking daily physical activity levels or specific occupational titles, this observed link must be interpreted with caution, as these unmeasured lifestyle behaviors could confound the relationship between educational background and joint pain reporting.

Our data also add to the growing body of survivorship literature showing that age remains a predictor of endocrine therapy-related vasomotor and sexual symptoms in univariate testing, despite all patients being postmenopausal, either naturally or induced. Younger patients have higher baseline sexual activity, and greater psychosocial distress related to body image and fertility [8]. Consequently, they may be more likely to experience vasomotor and sexual symptoms. Although age did not remain significant in our multivariate model, its robust univariate association aligns with existing evidence and reinforces the importance of age-tailored symptom management strategies independent of induced or natural menopause.

This study had several limitations. First, the cross-sectional design precludes causal inferences and limits the ability to capture temporal fluctuations in social media behavior and in symptom trajectories. Second, both exposure variables and clinical symptoms were assessed using self-reported tools, which may have introduced recall or social desirability bias. Third, unmeasured confounders such as baseline psychiatric diagnoses, coping styles, personality traits, pain thresholds, sleep disorders, or concomitant medications may influence symptom perception and were not captured in the current dataset. Moreover, we excluded those who do not use any social media platforms since they were ineligible to be included in the main surveys of this study which may have resulted a patient selection-bias. The major strength of this study was its unique and previously unexplored intersection in breast cancer survivorship by evaluating whether social media behaviors are associated with endocrine therapy-related vasomotor/sexual and musculoskeletal symptoms. The inclusion of two validated social media instruments (BSMAS and SMUS) enhanced the methodological rigor. The relatively homogeneous cohort of early-stage hormone receptor-positive survivors receiving contemporary endocrine therapy reduces clinical heterogeneity and allows for a clearer interpretation of symptom patterns.

## 5. Conclusions

Our findings demonstrate that among active social media users, varying intensities of unstructured digital exposure do not independently predict physical symptom burden. These within-user comparisons indicate that unstructured social media use may not be a clinically useful screening parameter for the endocrine therapy-related physical symptom burden. In routine care, clinical assessment should continue to prioritize established determinants such as patient age and educational background. Accordingly, symptom management pathways should focus on age- and menopause-tailored counseling to address the subjective burden of endocrine toxicities, rather than generic inquiries about social media use. From a broader research perspective, future investigative efforts might shift away from evaluating non-specific, unstructured social media environments. Instead, future prospective trials could explore whether structured digital therapeutics, such as tailored symptom-monitoring and adherence platforms, can provide measurable clinical benefits for endocrine toxicity relief and compliance.

## Figures and Tables

**Table 1:** Study Population Characteristics.

(*n* = 153)	*n*	%
**Age (years)**		
≤50	61	39.9
>50	92	60.1
**Sex**		
Female	153	100
**ECOG PS**		
0–1	153	100
**Marital Status**		
Married	112	73.2
Single	41	26.8
**Educational Status**		
Primary/Elementary School	79	51.6
High School/University	74	48.4
**Work Status**		
Working	37	24.2
Not Working	116	75.8
**Menopausal Status**		
Postmenopausal	97	63.4
Premenopausal	56	36.6
**Hormonal Therapy**		
Aromatase Inhibitor	97	63.4
Tamoxifen + OFS	56	36.6
**HER2 Status**		
Negative	113	73.9
Positive	40	26.1
T Stage *		
T1–2	130	85.5
T3–4	22	14.5
Nodal Status *		
Negative	68	44.7
Positive	84	55.3
**Chemotherapy History ¶**		
No	19	12.6
Yes	132	87.4

*: TNM stage of the primary tumor was missing for one patient; ¶: Chemotherapy data for two patients were missing; ECOG PS, Eastern Cooperative Oncology Study Group Performance Status; OFS, Ovarian Function Suppression.

**Table 2:** Univariate and Multivariate Analyses for Vasomotor/Sexual Symptom Score.

	Univariate Analyses		Multivariate Analyses	
		*p* Value	B (95% CI)	*p* Value
**Age**	**r = −0.360**	**<0.001**	−0.25 (−0.49–0.00)	0.051
**BSMAS Score**	**r = 0.194**	**0.017**	0.15 (−0.13–0.43)	0.298
**SMUS Score**	r = 0.083	0.310	−0.06 (−0.31–0.19)	0.623
**Marital Status** (mean ± SD)		0.099		0.085
Married	3.03 ± 0.10		Ref.	
Single	2.71 ± 0.17		−0.33 (−0.71–0.05)	
**Educational Status** (mean ± SD)		0.051		0.338
Primary/Elementary School	2.79 ± 0.12		Ref.	
High School/University	3.13 ± 0.14		0.17 (−0.18–0.53)	
**Work Status**		0.052		0.480
Working	3.25 ± 1.79		Ref.	
Not Working	2.86 ± 0.10		−0.15 (−0.58–0.27)	
**HER2 Status** (mean ± SD)		0.062		0.537
Negative	3.01 ± 0.10		Ref.	
Positive	2.87 ± 0.17		−0.12 (−0.52–0.27)	
**Hormonal Therapy** (mean ± SD)		**<0.001**		0.272
Tamoxifen + OFS	3.39 ± 0.12		Ref.	
Aromatase Inhibitor	2.71 ± 0.11		−0.26 (−0.72–0.20)	
**Chemotherapy History** (mean ± SD)		0.134		0.077
No	2.55 ± 0.24		Ref.	
Yes	3.02 ± 0.10		0.48 (−0.05–1.02)	
**Nodal Status** (mean ± SD)		0.817		
Negative	2.97 ± 0.13			
Positive	2.96 ± 0.12			

**B**, Unstandardised Regression Coefficient; **BSMAS**, Bergen Social Media Addiction Scale; **CI**, Confidence Interval; **Ref.**, Reference; **SMUS**, Social Media Use Scale.

**Table 3:** Univariate and Multivariate Analyses for Musculoskeletal Pain Score.

	Univariate Analyses		Multivariate Analyses	
		*p* Value	OR (95% CI)	*p* Value
**Age**	**r = −0.185**	**0.022**	0.98 (0.95–1.01)	0.149
**BSMAS Score**	r = 0.053	0.515	0.99 (0.61–1.60)	0.953
**SMUS Score**	r = 0.064	0.434	1.02 (0.67–1.55)	0.935
**Marital Status** (mean ± SD)		0.679		
Married	3.19 ± 0.11			
Single	3.28 ± 0.20			
**Educational Status** (mean ± SD)		**0.026**		**0.031**
Primary/Elementary School	3.00 ± 0.14		Ref.	
High School/University	3.43 ± 0.13		1.96 (1.06–3.57)	
**Work Status** (mean ± SD)		0.538		
Working	3.31 ± 0.21			
Not Working	3.18 ± 0.11			
**HER2 Status** (mean ± SD)		0.452		
Negative	3.09 ± 0.12			
Positive	3.49 ± 0.15			
**Hormonal Therapy** (mean ± SD)		0.309		
Tamoxifen + OFS	3.34 ± 0.17			
Aromatase Inhibitor	3.11 ± 0.12			
**Chemotherapy History** (mean ± SD)		0.255		0.237
No	2.83 ± 0.29		Ref.	
Yes	3.26 ± 0.10		1.70 (0.70–4.17)	
**Nodal Status** (mean ± SD)		0.966		
Negative	3.18 ± 0.12			
Positive	3.18 ± 0.14			

**BSMAS**, Bergen Social Media Addiction Scale; **CI**, Confidence Interval; **OR**, Odds Ratio; **Ref.**, Reference; **SMUS**, Social Media Use Scale.

## Data Availability

The data presented in this study are available on request from the corresponding author due to local ethical restrictions to protect subjects involved in the study.
